# Automatic Verification of the Gradient Table in Diffusion-Weighted MRI Based on Fiber Continuity

**DOI:** 10.1038/s41598-018-34940-4

**Published:** 2018-11-08

**Authors:** Iman Aganj

**Affiliations:** 1 Athinoula A. Martinos Center for Biomedical Imaging, Department of Radiology, Massachusetts General Hospital, Harvard Medical School, Charlestown, MA 02129 USA; 2 Computer Science and Artificial Intelligence Laboratory, Massachusetts Institute of Technology, Cambridge, MA 02139 USA

## Abstract

In diffusion-weighted magnetic resonance imaging (dMRI), the coordinate systems where the image and the diffusion gradients are represented may be inconsistent, thus impacting the quality of subsequent fiber tracking and connectivity analysis. Empirical verification of the reconstructed fiber orientations and subsequent correction of the gradient table (by permutation and flipping), both time-consuming tasks, are therefore often necessary. To save manual labor in studies involving dMRI, we introduce a new automatic gradient-table verification approach, which we propose to include in the dMRI processing pipeline. To that end, we exploit the concept of fiber continuity – the assumption that, in the fibrous tissue (such as the brain white matter), fiber bundles vary smoothly along their own orientations. Our tractography-free method tries all possible permutation and flip configurations of the gradient table, and in each case, assesses the consistency of the reconstructed fiber orientations with fiber continuity. Our algorithm then suggests the correct gradient table by choosing the configuration with the most consistent fiber orientations. We validated our method in 185 experiments on human brain dMRI data form three public data sources. The proposed algorithm identified the correct permutation and flip configuration for the gradient table in all the experiments.

## Introduction

By quantifying the diffusion of water molecules in the tissue, diffusion-weighted magnetic resonance imaging (dMRI) has pushed the boundaries of obtainable information from MRI to include the tissue’s microarchitecture. Structural connectivity of the central nervous system can be quantified by dMRI through neural fiber tracking (aka tractography)^1^, which takes advantage of the conventional MRI data augmented by fiber orientation information.

**Figure 1:**
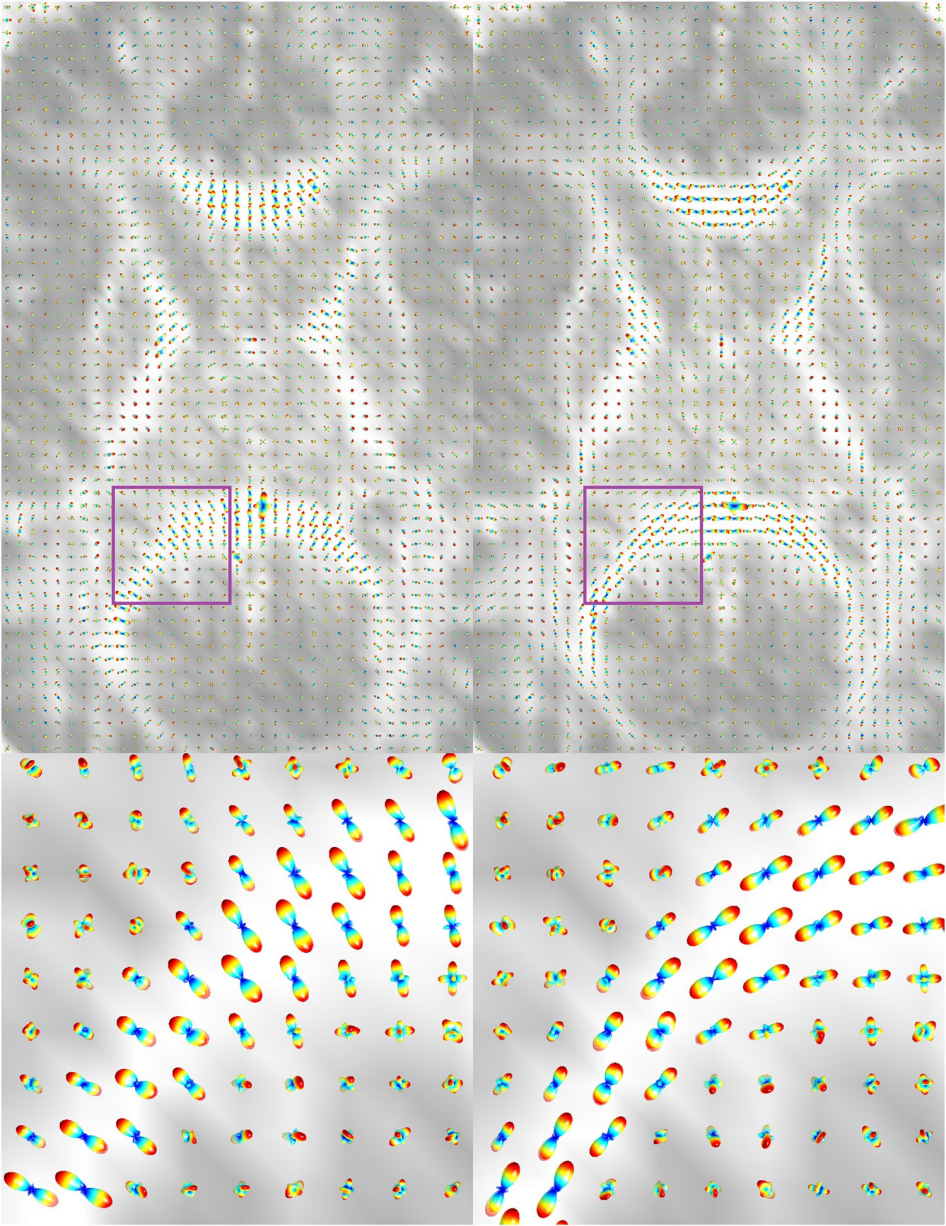
Axial view of the ODFs reconstructed from the Aarhus University dataset, overlaid on the GFA map. *Left:* The ODFs reconstructed with the original gradient table show an artificial 90° rotation about the *z*-axis. *Right:* The gradient table was corrected by swapping the *x*- and *y*-axes followed by flipping the *y*-axis.

To combine the spatial and orientational information obtained from dMRI, as is required e.g. in tractography, the location of macroscopic voxels and the spatial pattern of microscopic diffusion must be both represented in the same coordinate system. In practice, however, the directions of the gradients applied during the dMRI session, which are later provided to the user, may not always be in the image coordinate system. This misalignment leads to incorrect quantification of fiber orientations^2^. Depending on the scanner, the imaging protocol, and the software used^3–5^, one may need to permute the axes and/or flip an axis in the gradient table, to make the data usable (see Fig. 1 for an example). This is particularly important in the analysis of heterogeneous (e.g., multi-institutional) dMRI datasets that have been acquired on different scanners with different protocols. The required permutation and flipping of the gradient table often need to be figured out manually. This is a tedious task that involves trying different permutation and flip combinations and visually inspecting the resulting orientation distribution functions (ODFs)^6,7^ or tracts^1^, mainly in highly anisotropic regions such as the corpus callosum of the brain. Moreover, since the conventional color-coded orientation maps use absolute values of the vector components, they are not helpful in detecting fiber orientation errors resulted from gradient axis flipping^4^.

An automated method has been proposed^3^ to align the coordinate frame of the gradient orientations with that of the corresponding diffusion weighted images (considering flips, permutation, and small rotations due to angulation of the acquisition plane), which uses the average fiber trajectory length computed from whole brain fiber tractography as a metric. Another similar automatic tool^8^ suggests the appropriate flipping (though not the permutation) of the gradient table by counting the number of long tracts in the tractography results produced with each possible flip configuration. To ensure that repeated tractography in various alignment configurations does not result in a long runtime, algorithmic optimizations can be used in the whole-brain tractography routine^3^, or local tractography can be performed in a region of interest using simpler diffusion models.

In this work, we introduce a new automatic algorithm that, without using tractography, recommends the necessary permutation and flipping of the gradient table from the dMRI signal. We make use of the concept of fiber continuity^9,10^, which assumes that – due to the fibrous nature of the tissue – the value of the ODF in a particular orientation is likely to be spatially smooth in that orientation. We compute the fiber continuity error with all possible permutation and flip configurations for the gradient table, and then choose the configuration that produces the least error. We evaluate the performance of our method on human brain dMRI data from three public data sources.

## Methods

Let us denote the diffusion gradient table as *G*  := [*X Y Z*], where *X*_*N*×1_, *Y*_*N*×1_, and *Z*_*N*×1_ are vectors of the Cartesian coordinates of the *N* gradient directions. As an example, a permutation and flip configuration *T* that swaps the *y*- and *z*-axes and then flips the *y*-axis would transform the gradient table into $T(G)=[X\,-Z\,Y]$.

There are 6 possible permutations of the gradient table, for each of which 4 flip cases need to be tested, amounting to a total of 24 permutation-followed-by-flip configurations^3^. The 4 flip cases consist of the no-flip case, and the three cases where only one axis is flipped. Note that, because of the antipodal symmetry of the ODF, these 4 cases cover all possible flip configurations^8^. That is to say: 1) the case where all three axes are flipped is equivalent to the no-flip case, and 2) flipping exactly two (e.g., *x*- and *y*-) axes is equivalent to flipping all three axes (which has no effect thanks to symmetry) followed by flipping the remaining (i.e., *z*-) axis, which is already covered in the 4 cases.

For each configuration *T* out of the 24 possible ones, one can permute and flip the original gradient table *G*, and then reconstruct the ODF volume, ${\psi }_{T(G)}\,(\mathop{x}\limits^{\rightharpoonup },\,\hat{n})$, from the transformed gradient table *T*(*G*). For a water molecule initially located at voxel $\mathop{x}\limits^{\rightharpoonup }$, ${\psi }_{T(G)}\,(\mathop{x}\limits^{\rightharpoonup },\,\hat{n})$ is the orientational probability density function of diffusion of the molecule in the direction of unit vector $\hat{n}$ after a certain amount of time. The fiber continuity error^9,10^ for the configuration *T* can then be computed as:

1 $${\epsilon }(T)=\mathop{\int }\limits_{{\rm{\Omega }}}\mathop{\int }\limits_{{{\mathbb{S}}}^{2}}{(\hat{n}\cdot {\nabla }_{\mathop{x}\limits^{\rightharpoonup }}{\psi }_{T(G)}(\mathop{x}\limits^{\rightharpoonup },\hat{n}))}^{2}\,{\rm{d}}\hat{n}\,{\rm{d}}\mathop{x}\limits^{\rightharpoonup },$$

where ${\rm{\Omega }}\subseteq {{\mathbb{R}}}^{3}$ is the mask of the fibrous tissue (e.g., brain white matter), ${{\mathbb{S}}}^{2}$ is the unit sphere, and ${\nabla }_{\mathop{x}\limits^{\rightharpoonup }}$ is the gradient with respect to $\mathop{x}\limits^{\rightharpoonup }$.

Basically, a fiber bundle is expected to change smoothly (have a small directional derivative) along itself, thus contribute to ${\epsilon }$ only negligibly^9,10^. Fibers that change or end abruptly, on the other hand, contribute largely to ${\epsilon }$. Our underlying hypothesis is that the latter phenomenon should be rarely observed in the reconstructed ODFs of a fibrous tissue, unless a source of error – here misalignment of the gradient and image coordinate systems – affects the diffusion orientations. Therefore, as the output of the algorithm, we ultimately choose the permutation and flip configuration T^*^ that minimizes ${\epsilon }$:

2 $${T}^{\ast }=\mathop{{\rm{argmin}}}\limits_{T}\,{\epsilon }(T).$$

Note that, to ensure that other isotropic regions of the image do not add noise to ${\epsilon }$, it is critical to compute the spatial integral (${\rm{d}}\mathop{x}\limits^{\rightharpoonup }$) of Eq. (1) only inside the fibrous tissue (e.g., the brain white matter).

Since direct computation of *T* ^*^ would require reconstructing the ODFs 24 times, which would be time-consuming, we propose a different implementation scheme. Given that rotating and mirroring the gradient directions causes the reconstructed ODF to rotate and mirror similarly, one can verify that ${\psi }_{T(G)}\,(\mathop{x}\limits^{\rightharpoonup },\,\hat{n})={\psi }_{G}\,(\mathop{x}\limits^{\rightharpoonup },\,{T}^{-1}\,(\hat{n}))$. In other words, reconstructing an ODF with a transformed (permuted and flipped) gradient table *T*(*G*) and subsequently sampling it in the direction $\hat{n}$ is equivalent to reconstructing the ODF with the original gradient table *G* and subsequently sampling it in the direction ${T}^{-1}(\hat{n})$. Accordingly, we rewrite the expression for ${\epsilon }$ as:

3 $${\epsilon }(T)=\mathop{\int }\limits_{{\rm{\Omega }}}\mathop{\int }\limits_{{{\mathbb{S}}}^{2}}{(\hat{n}\cdot {\nabla }_{\mathop{x}\limits^{\rightharpoonup }}{\psi }_{G}(\mathop{x}\limits^{\rightharpoonup },{T}^{-1}(\hat{n})))}^{2}\,{\rm{d}}\hat{n}\,{\rm{d}}\mathop{x}\limits^{\rightharpoonup }.$$

Furthermore, since $T({{\mathbb{S}}}^{2})={{\mathbb{S}}}^{2}$, we make the change of variables $\hat{n}\leftarrow T(\hat{n})$, leading to:

4 $${\epsilon }(T)=\mathop{\int }\limits_{{\rm{\Omega }}}\mathop{\int }\limits_{{{\mathbb{S}}}^{2}}{(T(\hat{n})\cdot {\nabla }_{\mathop{x}\limits^{\rightharpoonup }}{\psi }_{G}(\mathop{x}\limits^{\rightharpoonup },\hat{n}))}^{2}\,{\rm{d}}\hat{n}\,{\rm{d}}\mathop{x}\limits^{\rightharpoonup }.$$

According to Eq. (4), we now need to reconstruct, sample, and compute the gradient of the ODFs only once with the original gradient table (instead of 24 times), which reduces the runtime of the algorithm considerably. We approximate the spherical integral (${\rm{d}}\hat{n}$) as a sum on the uniformly discretized unit sphere (although more sophisticated analytical approaches^11^ are also possible). After computing ${\nabla }_{\mathop{x}\limits^{\rightharpoonup }}{\psi }_{G}\,(\mathop{x}\limits^{\rightharpoonup },\,\hat{n})$ (once) for all $\hat{n}$ on the discrete spherical grid, we evaluate ${\epsilon }(T)$ for all 24 configurations *T*, and ultimately choose T^*^ as the minimizer of ${\epsilon }(T)$.

We arrived at Eq. (4) from Eq. (1) by relying on the fact that $T({{\mathbb{S}}}^{2})={{\mathbb{S}}}^{2}$, which is true for the continuous sphere. In the discrete case, however, permuting and flipping the uniform spherical grid will result in a uniform spherical grid that – due to the finite resolution – may not necessarily be identical to the original grid. Consequently, Eqs (1) and (4) may produce slightly different values as a result of discretization errors. Nonetheless, Eq. (4) is (not only computationally more efficient, but also) arguably more precise than Eq. (1). That is because Eq. (1) assesses the fiber continuity by sampling the ODFs in a slightly different set of orientations for each configuration *T* (due to the slight change in the spherical grid), thereby introducing an unwanted *T*-dependent variability (discretization artifact) in ${\epsilon }(T)$, whereas Eq. (4) uses a consistent set of ODF values across different configurations *T*, hence immunity to this specific discretization artifact.

## Results

We implemented our method in Matlab, and validated it in 185 experiments by analyzing existing de-identified human brain dMRI data from three public datasets, each containing images acquired with a number of different b-values (Table 1). We initially reconstructed the ODFs for each dataset in Matlab, and visually inspected them to see if it was necessary to permute the gradient table or flip one of its axes. This visual inspection served as the ground truth for our experiments. As we will see below, 15 experiments with varying b-values had misaligned original gradient tables. We then ran our gradient-table verification algorithm separately for each subject and each b-value, while computing ${\epsilon }$, for which we discretized the unit sphere into 23 uniformly distributed directions (corresponding to a resolution of 0.7 radians). For diffusion ODF reconstruction from a single shell, we employed q-ball imaging in constant solid angle^7^ and the real and symmetric spherical harmonic (SH) basis^12–14^, using the publicly available *CSA-ODF and Hough Tractography* Matlab package (www.nitrc.org/projects/csaodf-hough). For stability and robustness of the computed ${\epsilon }$, a less noisy ODF is preferable to a noisier one that has finer details. Hence, we used an SH order that was below the theoretical maximum SH order for each dataset.

**Table 1:** Summary of the datasets.

Data Source		Number of Experiments
Aarhus University^15^ 1 subject, b = 200, 400, …, 3000 s/mm², 33 directions		15
Human Connectome Project Harvard/MGH-USC Consortium^17,18^ 35 subjects	b = 1000 s/mm² 64 directions	35
	b = 3000 s/mm² 64 directions	35
	b = 5000 s/mm² 128 directions	35
	b = 10000 s/mm² 256 directions	35
Human Connectome Project WashU-UMN Consortium^20,21^ 10 subjects	b = 1000 s/mm² 90 directions	10
	b = 2000 s/mm² 90 directions	10
	b = 3000 s/mm² 90 directions	10
**Total**		**185**

For comparison, we also ran a publicly available implementation of (a subset of the functionalities of) a tractography-based algorithm^3^ on the datasets, to find the optimal permutation and flip aligning the gradient table to the image. This was done using the dwigradcheck command of the MRtrix3 package (www.mrtrix.org). Although this tractography-based approach^3^ additionally accounts for small rotations, its publicly available implementation does not account for such rotations (and neither does the proposed method).

As described below, both the proposed tractography-free approach and the existing tractography-based method succeeded to find the correct permutation and flip configuration in *all* the experiments.

### Public Human Brain Dataset – Aarhus University

We first tested our method on a publicly available human brain dMRI dataset from Aarhus University^15^. The dataset contained images acquired from a normal volunteer at 3 T at isotropic resolution of (2.5 mm)³, with the size 96 × 96 × 19. It included 15 different b-values, ranging from 200 to 3000 s/mm² (increments of 200), with 33 gradient directions for each b-value, in addition to a baseline (b = 0) image. We reconstructed and visually inspected the ODFs (from the included Matlab data files), and observed a 90° rotation about the *z*-axis, which could be corrected by swapping the *x*- and *y*-axes followed by flipping the *y*-axis in the gradient table. Figure 1 depicts the ODFs reconstructed with the SH order of 4 (left), and how they would look like after manual correction of the gradient table (right).

**Figure 2:**
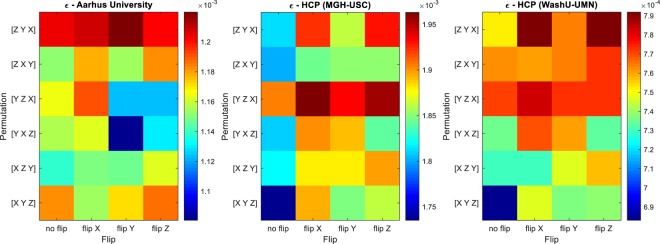
Fiber continuity error (${\epsilon }$) at each permutation and flip configuration, averaged across subjects and b-values, for the three human-brain data sources. Dark blue indicates the optimal configuration.

We ran our algorithm separately for each b-value. Given that the number of gradient directions were less than optimal for high-order fiber models^16^, we used a low SH order of 2, which still well captured the anisotropy in single-fiber voxels. We created a rough white-matter mask by only keeping voxels with mean apparent diffusion coefficient (ADC; average of $\mathrm{ln}\,({S}_{0}/S)/b$ over all directions, where *S*, *S*_0_, and *b* are the diffusion-weighted images, the non-diffusion-weighted image, and the b-value, respectively) smaller than 0.01 mm²/s, and the generalized fractional anisotropy (GFA)^6^ larger than 0.4. Note that scalars such as ADC and GFA are unaffected by permutation or flipping of the gradient directions. For all 15 b-values, both our method and the tractography-based method correctly identified the expected permutation and flip configuration (i.e., swapping the *x*- and *y*-axes, followed by flipping the *y*-axis). Figure 2 (left) shows the values of ${\epsilon }$ for all permutation and flip configurations, averaged across all b-values. As expected, the lowest value (dark blue) corresponds to the permutation $[Y\,X\,Z]$ followed by “flip Y”.

### Human Connectome Project – Harvard/MGH-USC Consortium

Next, we evaluated our method on the dMRI data of Harvard/MGH-USC Consortium of the Human Connectome Project^17,18^, consisting of brain images from 35 healthy adult participants. For each subject, data had been acquired at 3 T with four b-values of 1000, 3000, 5000, and 10000 s/mm², with 64, 64, 128, and 256 gradient directions, respectively, in addition to 40 images without diffusion weighting (b = 0). Images had isotropic resolution of (1.5 mm)³ and were of the size 140 × 140 × 96. Visual inspection revealed that no permutation or flipping was necessary for the gradient tables. (For one of the subjects, the images – but not the gradient table – were represented in a different frame. We transformed it so it conforms with the rest of the dataset.) We ran both methods for each subject and each b-value, and for our method, used the SH order of 4 and the white-matter mask produced by FreeSurfer^19^. Both the proposed and the tractography-based methods identified the correct gradient-table configuration for all the subjects and b-values. Figure 2 (middle) shows the value of ${\epsilon }$ for each permutation and flip configuration, averaged across all 35 subjects and 4 b-values. The lowest value (dark blue), as expected, corresponds to the case with no permutation $([X\,Y\,Z])$ and “no flip”.

### Human Connectome Project – WashU-UMN Consortium

Lastly, we performed similar experiments on brain images of 10 healthy adult participants from the dMRI dataset of the WashU-UMN Consortium of the Human Connectome Project^20,21^. For each subject, the dataset included images acquired at 3 T with three b-values of 1000, 2000, and 3000 s/mm². There were 90 gradient directions for each b-value, in addition to 18 baseline (b = 0) images. The voxel size was isotropic (1.25 mm)³, and the volume size was 145 × 174 × 145. Visual inspection again revealed that permutation and flipping of the gradient tables were not necessary. Similar to the previous subsection, we ran both methods for each subject and each b-value, and used the SH order of 4 and the white-matter mask provided by FreeSurfer^19^ for our method. Both our algorithm and the tractography-based method successfully identified the correct gradient-table configuration in all cases. Figure 2 (right) illustrates the average of ${\epsilon }$ across all 10 subjects and 3 b-values, for each permutation and flip configuration. The lowest value (dark blue) again correctly corresponds to the case with no permutation $([X\,Y\,Z])$ and “no flip”.

Due to its homogeneity, we chose this dataset to compare the runtimes of the two algorithms. We ran the two methods on a desktop PC with two Intel® Xeon® E5-2637 v3 3.50 GHz processors, each with four cores. We did not use any parallelization; nonetheless, both methods may have multithreaded their internal operations. The average runtime of the proposed algorithm was 23.0 ± (SEM) 0.1 s, in addition to 19.2 ± 0.4 s for loading the data from the disk. The average total runtime of the tractography-based^3^ method was 115.4 ± 1.7 s. It should be noted that the tractography-based method tested each permutation and flip configuration twice; once considering the coordinate system of the image and again considering that of the scanner, whereas our method only considered the former. Nevertheless, even half of the (computational) runtime of the tractography-based method was still longer than the runtime of our prototype.

## Discussion

In our validation on human brain datasets (Table 1), both the proposed tractography-free method and the existing tractography-based method^3^ succeeded to consistently identify the accurate permutation and flip configuration for the gradient table in all 185 experiments. Our method, however, had a shorter runtime.

Note that the diversity in software packages used to load and save the dMRI data is a major factor causing inconsistency between the loaded images and the gradient table. As opposed to the dwigradcheck command implemented in MRtrix3^3^, our public implementation is in Matlab, making it a good candidate for automatic inspection of dMRI data that is loaded for further analysis in Matlab.

Although our algorithm presents its output deterministically, other presentations of the results are also possible. For instance, instead of a single optimal permutation and flip configuration, all of them can be presented in the output, sorted with those having the smallest ${\epsilon }$ first. One can also take a probabilistic approach to compute a confidence value for the recommended configuration, based on the distribution of ${\epsilon }$ and appropriate priors.

To evaluate our method, we reconstructed the ODFs via q-ball imaging, which is used for high-angular-resolution diffusion images. Nevertheless, fiber orientations that are obtained through other acquisition or reconstruction schemes^16,22^ can be likewise used in our algorithm.

## Conclusions

A misrepresented gradient table can introduce large errors in fiber orientation estimation, thereby severely impacting the quality of subsequent tractography and connectivity analysis. Identifying any permutation or flipping of the gradient table is a crucial step in dMRI processing that is often done by trial and error, hence costing human labor. We have introduced a new tractography-free approach that alleviates this issue by automatically detecting the permutation and flipping that is necessary to correct the gradient table. When included in the dMRI processing pipeline, our method raises a red flag if it suspects an inconsistency between the image and gradient-table coordinate systems, and suggests the correct gradient table. Our evaluation on brain dMRI datasets revealed 100% accuracy for both our prototype and an existing tractography-based approach.

## Data Availability

The proposed method has been implemented in the checkGradTable function included in the publicly available *CSA-ODF and Hough Tractography* Matlab package (www.nitrc.org/projects/csaodf-hough)^7,23^. This function (like other functions in the toolbox) can optionally use a graphics processing unit (GPU). The MRtrix3 package^24^ that was used for comparison is also publicly available (www.mrtrix.org). The datasets analyzed in the current study are available to public from Aarhus University^15^ (10.5061/dryad.9bc43/1) and the Harvard/MGH-USC^17,18^ and the WashU-UMN^20,21^ consortia of the Human Connectome Project (https://neuroscienceblueprint.nih.gov/connectome-programs).
